# Microcrack and Porosity Development in Sealed Cement Mortars Measured with Micro-Computed Tomography

**DOI:** 10.3390/ma17133239

**Published:** 2024-07-02

**Authors:** Radek Ševčík, Irena Adámková, Michal Vopálenský, Pavel Martauz, Vít Šmilauer

**Affiliations:** 1 Institute of Theoretical and Applied Mechanics of the Czech Academy of Sciences, Centre Telč, Prosecká 809/76, 190 00 Prague, Czech Republic; adamkova@itam.cas.cz (I.A.); vopalensky@itam.cas.cz (M.V.); 2 Cement Plant Považská Cementáreň, a.s., 018 63 Ladce, Slovakia; martauz.p@pcla.sk; 3 Department of Mechanics, Faculty of Civil Engineering, Czech Technical University in Prague, Thákurova 7, 166 29 Prague, Czech Republic; vit.smilauer@cvut.cz

**Keywords:** microcracks, micro-computed tomography, sealed hydration, OPC, alkali-activated cement

## Abstract

For the first time, this paper explores the role of hydration kinetics on microcrack development in cement mortars using the μ-CT technique with a resolution of 2.2 µm. Three binders were tested: fine-grained ordinary Portland cement (OPC) with Blaine fineness of 391 m^2^/kg, coarse-grained OPC made from the same clinker with Blaine fineness of 273 m^2^/kg, and H-cement as a representative of the alkali-activated binder. It was found that most microcracks have a width in the range of 5–10 µm, increasing their occurrence with the progress of sealed hydration. While H-cement and coarse-grained OPC showed a comparable number of microcracks, fine-grained OPC exhibited more than twice the number of microcracks. In this sense, high hydration kinetics induce more microcracks, promoting later coalescence into visible cracks and disintegration of concrete at the end. Therefore, durable concrete with minimum microcracks should be derived from slow hydration kinetics or alkali-activation processes.

## 1. Introduction

Nowadays, Portland-based cements represent a fundamental material with an indispensable role in civil engineering construction that is used as a key component of concrete [1]. Cement-based materials are intensively used to build large-scale industrial, public, and private constructions [1]. The production of Portland cement, the most-produced man-made material, reached 4.1 billion tonnes in 2018 [2] and is burdened with high environmental impacts, especially during the calcination of limestone and fuel combustion [1,2]. The service life of concrete constructions is greatly affected by their susceptibility to the formation of cracks, leading to reduced mechanical performance, loss of concrete durability, increased permeability, reduced resistance to freeze-thaw cycles, and initialisation of the deterioration processes [3]. In general, several factors may affect the cracking during the lifetime of concrete, e.g., mineral admixtures [3], initial casting conditions [4], large variations of mechanical properties of applied constituents, and the role of the external environment [5,6,7]. A common characteristic of cracking development is the initial formation of non-visible (with the naked eye) microcracks with a width of a few micrometres and their gradual coalescence, leading later to the visible appearance of macrocracks in concrete structures [8,9]. Microcracks generally occur inside the cement paste, in the transition zones between the cement paste and aggregates, and within aggregate particles [10].

Focusing on the early stages of cement hydration, microcracks are formed due to chemical volume shrinkage—a significant process for microcrack growth due to internal and/or external restrains [1]. The hydration of C_3_S causes approximately 9 vol. % of chemical shrinkage [11,12]. The kinetics of chemical shrinkage plays a significant role in visible crack formation; a shorter time means lower stress relaxation and higher stress-inducing cracking. Experiments of 25 cements in externally restrained ring shrinkage tests proved that slower hydration kinetics prolong cracking time and yield more crack-resistant binders [3,13]. The current trend of using fine-grained cement with high strength gain is advantageous for construction speed but also induces microcracks, which may coalesce into visible macrocracks and impair long-term performance [3,13].

Several approaches have been utilised to obtain knowledge about microcracks formation. Most often, two-dimensional (2D) techniques, namely optical and scanning electron microscopy, have been applied to visualise the microcracks in various types of concretes [14,15,16]. Only a few 2D characteristics may be obtained, such as the distribution, length, and width of cracks. Nonetheless, the possibility of reliable quantitative analysis is very limited [17,18]. Moreover, specific procedures such as cutting, drying, impregnations, and polishing must be used before sample observations which may lead to artefacts within the investigated microstructures [16]. To overcome these limitations, three-dimensional (3D) non-destructive observations are necessary [10,19]. In recent decades, X-ray micro-computed tomography (μ-CT) has been identified as a suitable technique allowing the visualisation of 3D structures of building materials in an entirely non-invasive fashion [4,10,20,21,22,23]. Thanks to the rapid development of computational power and software tools, μ-CT imaging enables the extraction of many quantitative parameters in 3D [20,24,25,26].

This paper uses the μ-CT technique to study cement mortars to visualise and quantitatively describe microcrack development and propagation within microstructures. Three different types of cement, namely fine-grained, coarse-grained, and hybrid, have been selected to prepare mortars with fine silica sand, and the evolution of porosity has been investigated after two and four weeks of sealed hydration under constant temperature. Several quantitative parameters, including total porosity and connectivity, have been extracted from the data collected in fully 3D non-invasive measurements.

## 2. Materials and Methods

Three types of cements were manufactured by cement plant Považská cementáreň a.s. in Ladce, Slovak Republic, particularly:

- Fine-grained Portland cement (FGC; *d*_10_ = 2 µm, *d*_50_ = 12 µm, *d*_90_ = 42 µm, $\bar{x}$ = 17 µm) corresponding to CEM I 42.5R, Blaine fineness 391 m^2^/kg [27].
- Coarse-grained Portland cement (CGC; *d*_10_ = 6 µm, *d*_50_ = 21 µm; *d*_90_ = 48 µm, $\bar{x}$ = 24 µm), corresponding to CEM I 32.5R, Blaine fineness 273 m^2^/kg [27].
- Hybrid cement, H-cement (HC; *d*_10_ = 2 µm, *d*_50_ = 9 µm; *d*_90_ = 24 µm, $\bar{x}$ = 11 µm) as a representative of alkali-activated material, Blaine fineness 697 m^2^/kg [28].

Each cement’s chemical and mineralogical compositions are summarised in Table 1 and Table 2, with basic properties listed in Table 3. Both Portland cements were ground from the same clinker.

Pure quartz sand (≥99 wt.%; Roth, Germany), having a particle size from 250 to 360 µm, was used to produce cement micromortars. The constant w/c ratios of 0.55 were maintained for all three types of mortars and were prepared according to the following procedure: samples were mixed and homogenised in a laboratory mixer Hobart (ITW, Troy, OH, USA) for 4 min in the volume of 250 mL. No low-pressure/vacuum procedures were used to remove entrapped air. The mortars were finally cast into plastic round-shaped vials with an inner diameter of 6 mm and vibrated for 1 min. The sealed samples were kept in a stable environment in a climate chamber set up to 20 ± 1 °C and were measured sealed after 2 and 4 weeks of sealed hydration.

Isothermal calorimetry was conducted using a TamAIR, an 8-channel calorimeter (TA Instruments, New Castle, DE, USA). Released heat was determined according to EN 196-11 Method A [29] at w/c = 0.40.

Autogenous shrinkage was measured on pastes according to ASTM 1698-19 [30] using w/c = 0.55, zeroing strain at the final set.

**Figure 1:**
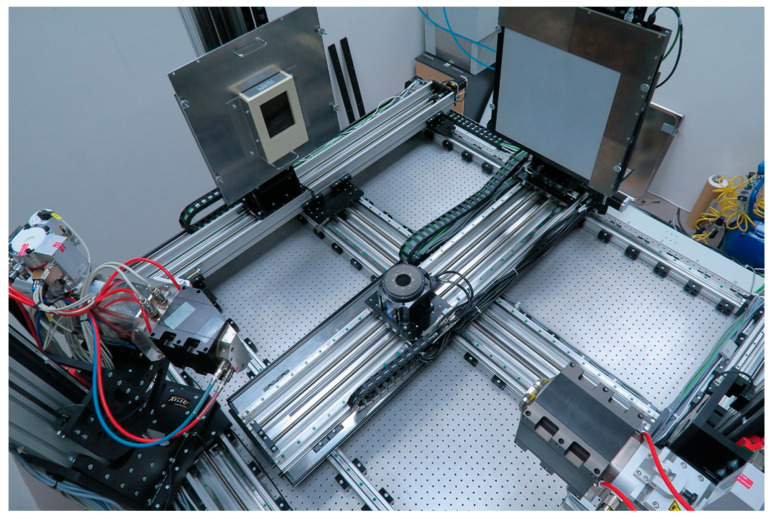
A photographic image of the tomograph, TORATOM.

Samples were scanned in an in-house developed experimental tomograph, TORATOM [31], depicted in Figure 1.

The device comprises two orthogonal imaging axes equipped with two independent X-ray sources. The preliminary measurement of the samples was made with a reflection-type XWT 240 SE tube (X-ray WorX, Garbsen, Germany), with the acceleration voltage of 220 kV and power of 50 W on the target; the beam was filtered with a 0.2 mm aluminium filter. An XRD 1611 flat panel detector (Varex Industrial, Salt Lake City, UT, USA) was used for imaging, with a well capacity of 0.25 pF and 2 × 2 binning, resulting in a pixel size of 200 µm. With the source-to-detector distance of 1080 mm and source-to-object distance of 24 mm, the volume element (voxel) size in the reconstruction volume was 4.44 µm. In total, 1920 angle steps over 360° were imaged with the exposure time of 400 ms; projection in each angle was averaged over 8 frames. The projections were corrected before reconstruction using a standard flat-field correction. Reconstruction was made using the VG Studio Max 2022.4.1 software (Volume Graphics, Heidelberg, Germany), employing the filtered back projection and FDK-algorithm.

Based on the reconstruction results, it was decided to try to increase the real resolution of the model. This was achieved with two measures: (a) using, as the X-ray source, a transmission-type XWT 160 TCHR tube (X-ray WorX, Garbsen, Germany) with an acceleration voltage of 150 kV and power at the target of 10 W, operated in microfocus mode; and (b) binning of pixels at the XRD 1611 detector was disabled. The XWT 160 tube in the described mode leads to a smaller radiation spot, which is an important factor in the achievable model resolution. With the binning disabled, the used detector provides a matrix of 4096 × 4096 pixels and a pixel size of 100 µm at the cost of considerably lower intensity in the individual pixels. However, the wider beam aperture of the XWT 160 tube compared to that of the XWT 240 tube allowed shortening the source-to-detector distance to 800 mm, increasing the intensity on the detector. The source-to-object distance was set to 17.6 mm, resulting in a voxel size of 2.2 µm in the reconstructed volume. In each scan, 1800 projection angles per 360° were imaged with the exposure time of 1800 ms and averaged over 2 frames. Before reconstruction, the projection images were corrected more extensively to reduce the beam-hardening artefact in the reconstruction, which was performed again in the VG Studio Max software.

The reconstructed two-dimensional slices were visualised using the Fiji 2.9.0 software [32]. Therefore, after adjusting brightness and contrast, each original dataset was cropped to an 8-bit volume of interest (VOI). The size of VOIs was set up to 900 × 900 × 600 (with voxel resolution 2.2 µm) according to the previous investigations showing that the dimensions of representative VOIs should be 2.0–2.5 times that of the largest aggregate particles [25,33]. The segmentation process, image filtering, and all analyses were conducted using Dragonfly 2022.1 software (Comet Technologies Canada Inc., Montréal, QC, Canada). To enhance void visibility, the median filter was applied. The inscribed diameter was used to determine the width of voids [34] and the maximum ferret diameter to calculate the lengths of voids. Connectivity calculations were done following the procedure described in [34] based on watershed segmentation and scanning labels presented in the layer or shell of a voxel in contact with each area. Each final pore network was created by a segmentation process based on the greyscale histogram.

## 3. Results and Discussion

**Figure 2:**
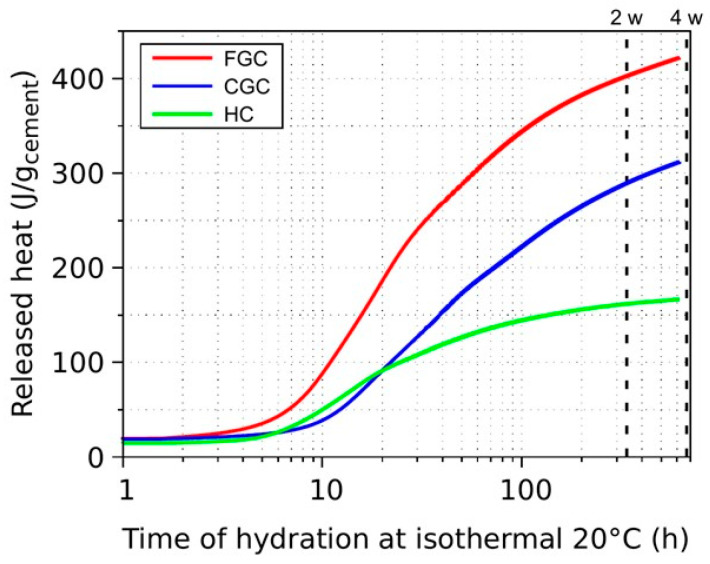
Released heat during hydration of three cements (FGC—fine-grained cement; CGC—coarse-grained cement; HC—hybrid cement).

Figure 2 shows the released heat during the hydration of the three cements. It is valuable to estimate the overall degree of hydration, capillary, and gel pore fractions using the standard model of Powers and Brownyard for cement paste [35]. The degree of hydration was calculated from the released heat divided by the potential hydration heat from Table 4. Volume fractions of capillary pores and gel pores used the following formulas:

(1) $$f_{capillary\;pores}=\frac{\frac{w}{c}-0.36DoH}{\frac{w}{c}+0.32},$$

(2) $$f_{gel\;pores}=\frac{0.19DoH}{\frac{w}{c}+0.32},$$

where DoH stands for degree of hydration. Open porosity accessible for water, quantifying total evaporable water, corresponds to the sum of the capillary and gel porosities.

The volume fractions of H-cement were approximated by a model of alkali activation of fly ash [36]. The water/cement ratio of 0.55 is close to the activator/solid ratio of 0.531. Corresponding degrees of reactions are summarized in Table 4 for 2 and 4 weeks. The main hydration product, N-A-S-H gel, has no characteristic porosity, making the distinction between capillary and gel pores arbitrary. Instead, helium-accessible open porosity is reported.

**Figure 3:**
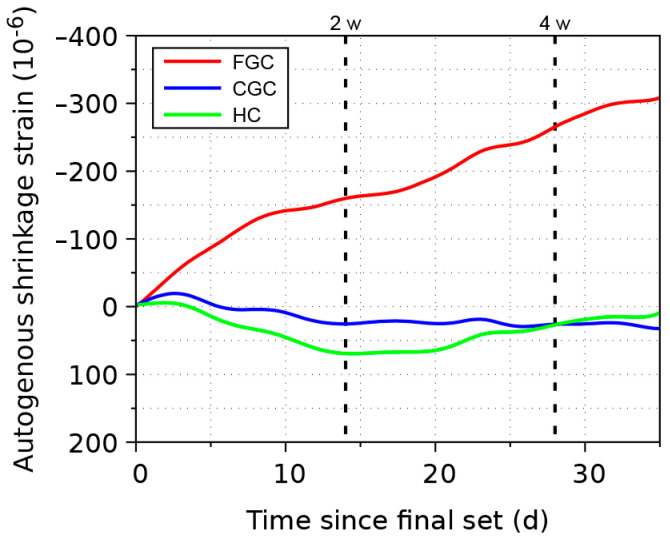
Autogenous shrinkage of three cements (FGC—fine-grained cement; CGC—coarse-grained cement; HC—hybrid cement).

The evolution of the autogenous shrinkage of pastes is summarized in Figure 3. Higher water demand due to faster hydration in FGC apparently leads to capillary tension and macroscopic shrinkage, attaining −265·10^−6^ at 4 weeks. CGC and HC show relatively small swelling. The state of microcracks remains unknown, since autogenous shrinkage is the summation of the expansive growth of hydrates and the effect of capillary tension as the first approximation. It is obvious that further restraining FGC by aggregates will cause microcracks due to internal restraint between the paste and aggregates.

A μCT resolution of 2.2 μm can identify well any entrapped-air voids in an approximately spherical shape, and a fraction of larger capillary pores and microcracks. Capillary porosity decreases due to hydration, while microcracks propagate due to ongoing chemical shrinkage. Separation between both groups is a delicate subject, requiring a set of morphology filters [25].

**Figure 4:**
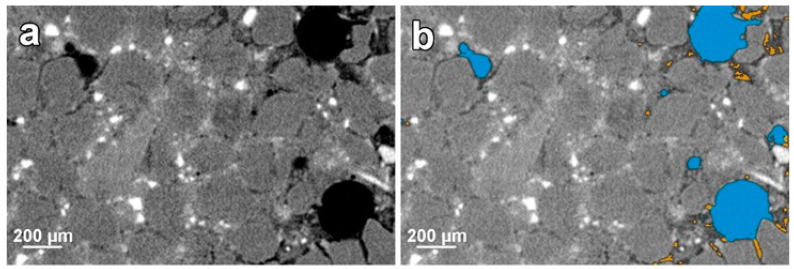
An example of an axial view of μ-CT slices inside the VOI (**a**) with voids indicated by black colour and (**b**) segmented entrapped-air voids (highlighted in blue colour) and formed microcracks (highlighted in orange colour) of a sample composed of fine-grained cement (FGC) aged for 4 weeks.

An example of the axial view of a selected slice of the VOI after the reconstruction and segmentation of a sample of CGC is depicted in Figure 4. The individual components presented in the cement mortar can be recognised due to their differences in attenuation of the X-ray beam because of their various densities and atomic numbers [37]. Thus, each component has its specific greyscale values [38]. The homogenous distribution of dark grey siliceous aggregate particles within the light grey to white (according to the abundance of particular phases inside the cement) cement matrix is visible (Figure 4a). Due to the high contrast between solid particles and voids (in black colour), voids can be extracted from the mortar`s components as visualised in Figure 4b.

**Figure 5:**
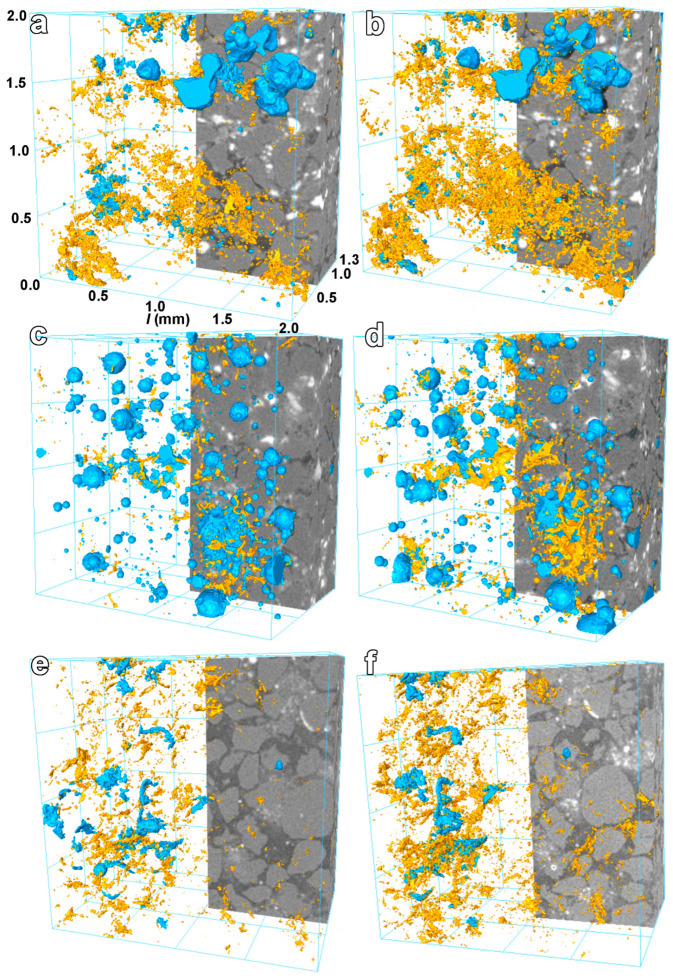
Graphical visualisation of the evolution of entrapped air (blue) and microcracks (yellow) in similar VOIs of samples aged for 2 (on the left) and 4 weeks (on the right) composed of fine-grained cement (FGC; **a**,**b**), coarse-grained cement (CGC; **c**,**d**), and hybrid cement (HC; **e**,**f**). The dimensions of the VOIs are indicated in (**a**).

In addition, different voids can be separated due to their high morphological variations, mainly larger spherulitic entrapped-air voids and irregularly shaped smaller voids corresponding to the microcracks, highlighted in blue and orange colour, respectively, in Figure 4b. The 3D views of extracted air voids and microcracks within the VOIs of the tested micromortars are shown in Figure 5. The 3D visualization confirmed the findings from the limited 2D observations that most of the microcracks were present in close contact with entrapped-air voids and aggregate particles and distributed within the whole of the VOIs, suggesting their formation due to autogenous shrinkage, in agreement with previously published observations [25]. In general, the increased formation of microcracks around aggregate particles in so-called interfacial transition zones (ITZs) is explained in Portland-based cements by the wall effect with locally increased porosity and the presence of complex microstructure, including zones with large portlandite and ettringite crystals [39] connected to the aggregates through the micrometric layer of the calcium silicate hydrate (C-S-H) layers. For this reason, ITZ usually represents the weakest part of a mortar for the initial formation of microcracks and their elongation and subsequent propagation [40]. In contrast, alkali-activated systems form a 3D polymer network with no characteristic gel porosity [36]. Such materials generally exhibit better bonding with aggregates [41], reflected in their lower microcrack formation.

All tested cements showed an increased number of detected microcracks when the reaction proceeded from 2 to 4 weeks (in Figure 5, on the right). This experiment testifies that volume shrinkage associated with chemical shrinkage presents a major driving force. Mac et al. [25] observed the same kinetic trend for μ-CT data collected for silica fume concrete. The results of the quantitative analysis are summarized in Table 5 and Table 6.

**Figure 6:**
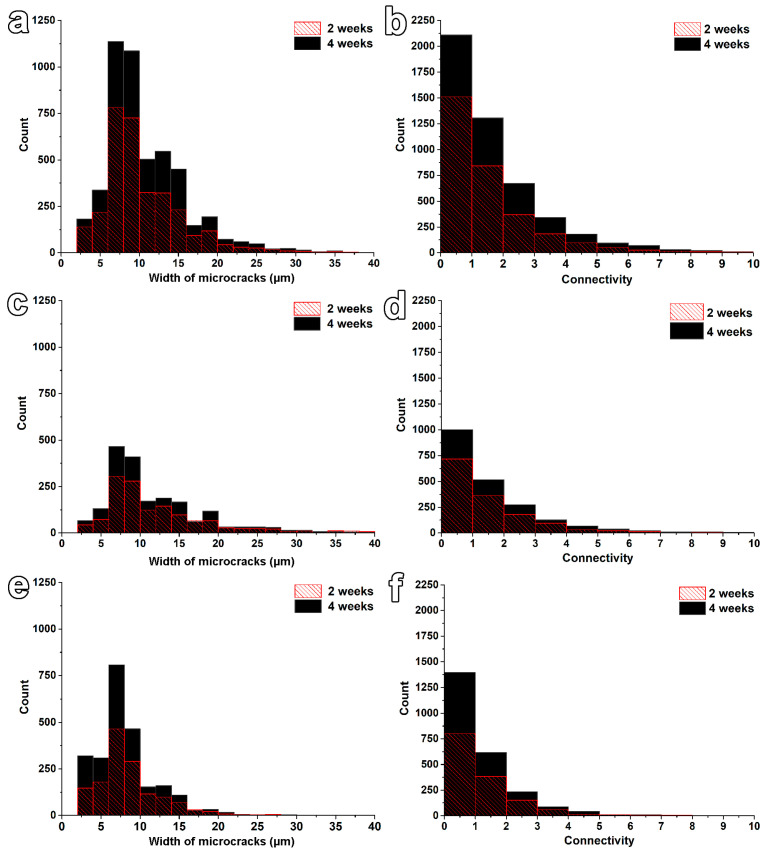
The quantitative evaluation of microcracks’ formation (on the left) and their connectivity development during sealed hydration of mortars composed of fine-grained cement (FGC; **a**,**b**), coarse-grained cement (CGC; **c**,**d**), and hybrid cement (HC; **e**,**f**).

The total number of microcracks was the highest for the sample of FGC for both curing times. If the values calculated for 2 weeks are compared, the CGC and HC samples displayed around half of the microcracks. In contrast with results obtained by Mac et al. [25], the total number of microcracks increased with hydration time by approximately 84, 48, and 53% for the samples of FGC, CGC, and HC, respectively. Higher amounts of detected microcracks in this work could be ascribed to the higher voxel resolution of recent μ-CT scanning, i.e., 2.2 µm instead of the previously used 15.5 µm [25]. For this reason, not only the interconnection and propagation of cracks but also the formation of new cracks of a size of a few microns could be detected, even if an even higher resolution is needed to see capillary and gel pores inside ITZs [40]. The average ($\bar{x})$ and median (*M*) values of microcracks’ widths and lengths were found to be comparable for all micromortars. Figure 6 (on the left) graphically shows the development of microcracks’ widths. Most of their widths were found to be in the range of a few microns (due to the reached voxel resolution) up to 40 µm. Most microcracks were identified within the 5–10 µm range. Such values agree with the observations of Lura et al. [14] for cement paste with silica fume. Notably, the aggregates’ size also plays an essential role in microcracks’ formation and their dimensions [42]. As reported by Yio et al. [18], replacing aggregates with a maximum size of 5 instead of 20 mm led to developing a lower volume fraction of microcracks with smaller dimensions during autogenous shrinkage. With increasing hydration times, more microcracks were identified, and their widths were shifted to higher values due to the expected microcrack elongation, propagation, and coalescence. The higher formation of microcracks over time was also identified according to their determined total lengths. Between 2 and 4 weeks of hydration, the total lengths of microcracks increased by a factor of 2.0, 2.5, and 2.0 for FCC, CGC, and HC, respectively. Overall, FCC displayed the most developed microcrack network.

The calculated total porosity values visible to μ-CT, including the extracted porosity for only air voids and microcracks, are listed in Table 6.

The sum of the volume fractions of the entrapped air and microcracks of HC was at least five times lower compared to the samples composed of Portland cement (FGC and CGC). The explanation can be found in their dissimilar compositions, as Table 1 and Table 2 indicate, and in the different behaviour of alkali-activated materials [43]. The majority of voids belonged to entrapped air, as a result of the high water/binder ratio of 0.55 used for the production of the Portland cement mortars.

The only exception was HC cured for 4 weeks. In the FGC and CGC samples, the volume fractions of entrapped air were identified to be around 1.4 and 2.3 percent. In a previous study [25], 2.1 vol.% of entrapped air voids was reported; however, lower water/binder ratios were adopted.

The minor differences in the air voids’ porosity values for samples aged for 2 and 4 weeks correspond to the slightly shifted VOIs, as depicted in Figure 5. Looking closely at the microcracks’ kinetic development, the increments were detected in all tested systems in line with previous μ-CT observations of microcrack formation [25]. Namely, the volume fractions of microcracks increased by 2.4, 3.6, and 1.6 times between 2 and 4 weeks for the FGC, CGC, and HC micromortars, respectively. Generally, the FGC had the highest tendency to the formation of microcracks.

**Figure 7:**
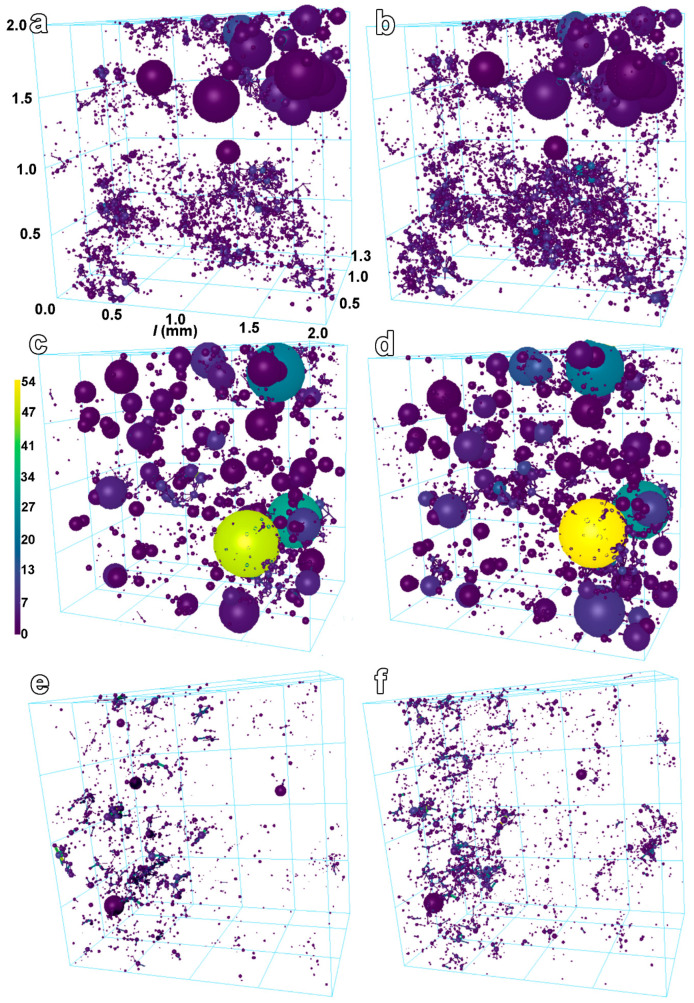
The graphical visualisation of simplified connectivity development of samples aged for 2 (on the left) and 4 weeks (on the right) composed of fine-grained cement (FGC; **a**,**b**), coarse-grained cement (CGC; **c**,**d**), and hybrid cement (HC; **e**,**f**). The size of the spheres is proportional to the pore volume. The dimensions of VOIs and colour scale bar characterising the connectivity are visualised in (**a**) and (**c**), respectively.

Connectivity results, displayed in Figure 7, show that entrapped-air voids represent parts with the highest interconnections (see Figure 7), especially in the CGC. The interconnection of only microcracks was lower by around one order of magnitude compared to the interconnection of air voids with microcracks. In both these types, increased interconnection was identified with prolonged curing times for all tested samples, as depicted in Figure 6 and Figure 7 (on the right). The connectivity was calculated to increase from HC to CGC and, finally, FGC. The maximum values of connectivity reported in Table 7 are ascribed to the highly interconnected areas in contact with air voids. The interconnection of solely microcracks was identified to be mainly in the range from 0 (isolated microcracks) to 6. As discussed above, the increasing number of isolated microcracks confirmed the formation of new cracks between 2 and 4 weeks of sealed hydration. The comparison with literature values is very limited. The same procedure for the calculation of connectivity from μ-CT was used to visualise and quantify significant changes in the microstructure of sandstones induced by the application of polymeric dispersions [44]. Previous works calculated connectivity in cement-based materials differently, as the volumetric ratio of the largest connected microcrack to all microcracks, and the interconnection was identified to be in the range of 18.0 to 89.1 and 3.33 and 54.2 in the dependency on types of concrete [25] and used w/b ratios [18], respectively. Interestingly, it was reported that the continuous in-situ 3D microcrack connectivity analysis did not reveal significant changes in samples aged from 1 to 28 days [25]. In addition, the connectivity in the previous work [25] was higher for the smaller cast samples than the cored samples, suggesting a size effect and increased physical restraint of smaller moulds. It should be noted that the higher microcracking caused by autogenous shrinkage does not automatically lead to the increased mass transport properties, as pinpointed in [18].

This study successfully visualised and quantitatively described the development of microcracks and their interconnectivity during sealed hydration and highlighted the susceptibility of different cementitious binders to their formation and propagation. It was shown that the micromortar composed of fine-grained Portland cement (FGC) had an increased tendency to form microcracks at the studied hydration intervals compared with coarse-grained Portland cement of identical chemical and mineralogical composition. The observed and quantitatively described microcracking formation may help to understand the hydration of cement-based composites better and select suitable cements for long-life concrete structures.

P.K. Mehta [9] and R.W. Burrows [11] have presented already decades ago the importance of minimal microcrack formation for durable concrete. They described concrete disintegration due to the growth and coalescence of microcracks. A driving force is necessary to stimulate crack growth, which is commonly provided by fluctuating temperature or humidity conditions in outdoor environments. At a certain point, the stability condition of parallel cracks requires the closure of every n^th^ crack while opening and growing neighbourhood cracks. Such a situation repeats during the crack growth [8] and describes the transition of invisible microcracks to visible cracks. In 1928, P. H. Bates from the National Bureau of Standards wrote “It is unfortunate that the producers of these new cements cannot see the value of obtaining critical data other than strength, particularly when it has been so well established that strength is not a criterion of durability of normal cements in concrete”.

## 4. Conclusions

The X-ray micro-computed tomography (μ-CT) was used to characterize entrapped air and microcrack formation during sealed hydration. Three cements were tested with the following conclusions:

1. Microcrack width falls in the range of 0–40 μm after 2 weeks of hydration, where 5–10 μm is the most representative region for all three tested cements.
2. The volume of the microcracks increased 1.6–3.6× between 2 weeks and 4 weeks of hydration. Correspondingly, the number of microcracks increased 1.8–2.1× and their total length 1.9–2.5×.
3. Fine-grained OPC with a Blaine fineness of 391 m^2^/kg produced the highest number of microcracks, approximately twice as much as the coarse-grained cement or alkali-activated H-cement. Under environmental driving forces, such as temperature or moisture changes, microcracks will tend to grow, propagate, and coalesce, changing invisible microcracks to visible cracks. The consequences are reduced lifetime, lower durability, or concrete disintegration. The current pressure on construction schedules with fine-grained cements presents potential long-term risks.

## Figures and Tables

**Table 1:** Chemical composition of used cements.

Formula	*c* (wt.%)		
	FGC	CGC	HC
CaO	64.6	64.8	33.8
SiO_2_	19.2	19.3	33.3
Al_2_O_3_	5.3	5.2	14.0
Fe_2_O_3_	3.4	3.6	5.4
MgO	1.8	1.6	1.8
K_2_O	1.2	1.2	2.0
Na_2_O	0.1	0.1	3.6
SO_3_	3.2	3.0	5.4
Cl^−^	<0.1	<0.1	<0.1

**Table 2:** Mineralogical composition of used cements.

Formula	Mineral	*c* (wt.%)		
		FGC	CGC	HC
C_2_S	Belite	4.6	5.9	4.7
C_3_S	Alite	73.3	70.7	31.0
C_3_A	Aluminate	5.6	3.1	2.4
C_4_AF	Brownmillerite	10.4	8.7	5.2
CS	Anhydrite	3.3	4.7	-
CSH_2_	Gypsum	1.8	2.6	-
C	Lime	-	1.3	-
S	Quartz	0.5	0.7	7.5
AS_2_	Mullite	-	-	10.1
NS	Thenardite	-	-	8.3
MC	Magnesite	-	-	2.2
Amorphous	Amorphous	0.2	2.2	28.7

**Table 3:** Overview of basic properties of used cements.

Property	Cement Type		
	FGC	CGC	HC
Blaine fineness (m^2^/kg)	391	273	697
Compressive strength—2 d (MPa)	32.6	11.9	14.8
Compressive strength—28 d (MPa)	58.0	37.9	32.2
Flexular strength—2 d (MPa)	6.7	3.3	3.5
Flexular strength—28 d (MPa)	9.0	6.8	5.4
Potential hydration heat (J/g)	545	545	-

**Table 4:** Microstructural details of three cements in terms of predicted volume fractions.

Descriptor	*t* (Weeks)	Cement Type		
		FGC	CGC	HC
Degree of reaction	2	0.74	0.53	0.15
	4	0.75	0.58	0.20
Capillary porosity	2	0.33	0.41	
	4	0.31	0.39	
Gel porosity	2	0.16	0.12	
	4	0.17	0.13	
Open porosity	2	0.49 (water)	0.53 (water)	0.39 (helium)
	4	0.48 (water)	0.52 (water)	0.37 (helium)

**Table 5:** The summarization of selected statistical values determined for microcracks, characterizing their widths and lengths (FGC—fine-grained cement mortar; CGC—coarse-grained cement mortar; HC—hybrid cement mortar). Numbers in brackets show calculated standard deviations.

Sample	*t* (Weeks)	Microcracks’ Number	Microcracks’ Width (µm)			Microcracks’ Length (µm)		
			Average	Median	Maximum	Average	Median	Total Length
FGC	2	2555	9 (4)	8.6	34.5	31(18)	25.0	23,270
	4	4713	10 (4)	9.4	42.3	33(20)	27.4	46,944
CGC	2	847	8 (3)	8.6	21.1	27(12)	23.7	7133
	4	1739	10 (5)	9.4	42.3	30(17)	24.6	17,694
HC	2	1189	7 (3)	6.0	22.6	30(17)	24.6	8793
	4	2228	7 (3)	6.0	29.4	30(18)	24.8	16,851

**Table 6:** Volume fractions of entrapped air, microcracks, and summation (FGC—fine-grained cement mortar; CGC—coarse-grained cement mortar; HC—hybrid cement mortar).

Sample	*t* (Weeks)	*Φ* tot (vol.%)	*Φ* Entrapped Air (vol.%)	*Φ* Microcracks (vol.%)
FGC	2	1.91	1.41	0.50
	4	2.56	1.36	1.20
CGC	2	2.31	2.21	0.10
	4	2.82	2.46	0.36
HC	2	0.32	0.15	0.17
	4	0.49	0.18	0.27

**Table 7:** The summarisation of selected total connectivity statistical characteristics (FGC—fine-grained cement mortar; CGC—coarse-grained cement mortar; HC—hybrid cement mortar). Numbers in brackets show calculated standard deviations.

Sample	*t* (Weeks)	Average	Median	Minimum	Maximum
FGC	2	1.1(1.6)	1	0	17
	4	1.3(1.8)	1	0	23
CGC	2	1.1(2.2)	1	0	47
	4	1.2(2.2)	1	0	54
HC	2	0.8(1.2)	0	0	10
	4	0.7(1.3)	0	0	13

## Data Availability

The data presented in this study are available on demand.
